# Towards multiple ontologies in science mapping. A tribute to Loet Leydesdorff

**DOI:** 10.1007/s11192-025-05323-0

**Published:** 2025-04-25

**Authors:** Ismael Rafols

**Affiliations:** 1 INGENIO (CSIC-UPV), Universitat Politècnica de València, València, Spain; 2 UNESCO Chair On Diversity and Inclusion in Global Science, Centre for Science and Technology Studies (CWTS), Leiden University, Leiden, The Netherlands; 3 SPRU (Science Policy Research Unit), University of Sussex, Brighton, England

**Keywords:** Science maps, Classification, Ontologies

## Abstract

This article reviews Loet Leydesdorff’s contributions to science mapping. It explains how over the years, his mapping techniques evolved from journal mapping to global maps of science and finally towards interactive interfaces portraying multiple classifications and ontologies. It then critically reviews the challenges faced by current approaches to science mapping, which implicitly assume a ‘natural’ epistemic structure, with examples from two recent case studies. We observe that bottom-up algorithmic approaches, either based on citation or semantic approaches, lack conceptual consistency regarding the type of categories used: in a same classification a category captures methods, another one has materials, a third one contains empirical objects and a fourth is focused on theories, rather than having a single logic. I argue that science mapping would produce more useful representations by using ontologies based on a single logic that aligns with the particular conceptual needs of the analysis. Novel classification methods based on machine learning and language models hold promise to produce these tailored, question-driven ontologies.

## Introduction

“This book first arose out of a passage in Borges, out of the laughter that shattered, as I read the passage, all the familiar landmarks of my thought—*our* thought that bears the stamp of our age and our geography—breaking up all the ordered surfaces and all the planes with which we are accustomed to tame the wild profusion of existing things, and continuing long afterwards to disturb and threaten with collapse our age-old distinction between the Same and the Other. This passage quotes a ‘certain Chinese encyclopaedia’ in which it is written that ‘animals are divided into: (a) belonging to the Emperor, (b) embalmed, (c) tame, (d) suckling pigs, (e) sirens, (f) fabulous, (g) stray dogs, (h) included in the present classification, (i) frenzied, (j) innumerable, (k) drawn with a very fine camelhair brush, (l) *et cetera,* (m) having just broken the water pitcher, (n) that from a long way off look like flies’. In the wonderment of this taxonomy, the thing we apprehend in one great leap, the thing that, by means of the fable, is demonstrated as the exotic charm of another system of thought, is the limitation of our own, the stark impossibility of thinking *that*.” (Foucault, 2012)

‘What are the natural structural units of science? How are these structural units related to one another? What are the forces which determine these structural units and their interrelations? How does the structure of science change over time, both at a macro- and at a micro-level?’ (Leydesdorff, 1987, p. 294). These questions, with a focus on epistemic and communication (rather than social) dynamics, were central in Loet Leydesdorff’s thought (Leydesdorff, 1986, 1987).

In this article, I will seek to explore how Leydesdorff developed and used science mapping techniques to explore these questions. I will explain how his science mapping evolved from journal mapping to overlay maps and finally towards portraying multiple classifications and ontologies (Petersen et al., 2016; Rotolo et al., 2017). In contrast to Leydesdorff’s approach, I will argue that current science mapping suffers from the same affliction of the Chinese encyclopaedia that made Foucault burst out laughing (or is it Leydesdorff’s distinctive laughter reverberating?): a lack of recognition of the plurality of classifications and multiple ontologies that exist in science. My proposal is that science mapping should embrace these multiple ontologies in accordance with the specific questions it aims to address.

In this tribute to Loet Leydesdorff, I will try to honour his critical and provocative spirit and his engagement with theoretical and sociological considerations. First, I will explain how we came to work together for about ten years. Next, I will share Leydesdorff’s main contributions to science mapping. This will be followed by a critical appraisal of the state-of-the-art of science mapping, and its challenges, including insufficient empirical and theoretical engagement. Finally, I will propose that conceptual specificity on the logics of classifications and ontologies may improve the value of science mapping both for sociological research and for pragmatic science policy applications – and that novel language models offer the opportunity to do so.

## The encounter with a generous critical voice

In 2007 I was carrying out studies on inter- and multidisciplinarity in the ‘emergent field’ of nanotechnology (Rafols, 2007; Rafols & Meyer, 2007). I had previously worked in the Center for Nanobiotechnology at Cornell University, a front runner in the field, and was quite uncomfortable with bibliometric reports that presented nanotechnology as ‘one’ field and claimed that it was highly interdisciplinary. Working at the Department of Physics at Cornell, I had a first-hand experience of the disparate knowledge bases and practices across the different labs engaged in nanotechnology, and how difficult and infrequent cross-fertilization was. Scientometrics had to find a way of describing nanotechnology that captured large epistemic distances across areas, which local (conventional) article or journal citation maps failed to convey. That year, Kevin Boyack, Dick Klavans and Katy Börner presented a global map of science (the so called ‘backbone of science’) based on journal-to-journal citations in the conference of the International Society of Scientometrics and Informetrics (ISSI) (Boyack et al., 2005). This type of global map would offer the opportunity to show the locations of different nanotechnology areas – but it was not publicly available.

During the conference, I had the chance to discuss this global map with Leydesdorff. He worked regularly with the full Journal Citation Reports (JCR), which included journal to journal citations of all the Web of Science. However, we could not make a global journal network like Boyack’s because, at the time, it was computationally too demanding to run clustering, factor analysis or network visualisation over the more than 6000 journals that constituted the Science Citation Index database in 2006 (Leydesdorff & Rafols, 2009, p. 348). Moreover, Leydesdorff was very sceptical that a robust global map could be produced because it depended on many arbitrary methodological choices. During our discussion, we had the idea of using directly Web of Science (WoS) categories as disciplinary categories. Surprisingly, it turned out that the network of citation similarities across 250 subject categories produced a ‘nice’ global map of science with a sensible disciplinary structure (Leydesdorff & Rafols, 2009).

The success of this quick experiment led Leydesdorff to one of those working binges associated with intellectual excitement – so intense that were difficult for collaborators to follow. Before I could even think of it, he had already written the first paper of a series of collaborations on science mapping, focused on interdisciplinarity and emergent fields, that lasted almost ten years (Rafols et al., 2010, 2012; Rotolo et al., 2017).

At that time, Leydesdorff was a highly recognised professor, but rather than as a teacher or a master, he acted as a collaborator with an eager, sometimes frenetic interest to come up with and implement new ideas, sharing his knowledge quickly and openly. I have witnessed his generosity and his joy of supporting and working with young scholars, not only with me, but also with colleagues like Daniele Rotolo. I have to confess that as I came to know Leydesdorff better, I sometimes waited weeks or months before telling him about new ideas for joint work because I could not cope with his speed!

When I started collaborating with Leydesdorff in 2007, I was not aware of the degree to which our joint work was building on more than 20 years of efforts on science mapping – which of course had a major influence on the type of bibliometrics that colleagues and I conducted in SPRU (my institute at the University of Sussex) thereafter. It also took me some years to realise that Leydesdorff’s intellectual position was extremely rare, always in the margins, combining acid critiques with his light laugh.

Since the 2000s, after the closure of the department of *Science and Technology Dynamics*, he was a relatively lone professor with many (distant) collaborators, in spite of being recognised for his scientific contributions. Institutionally, he was a bit of an outcast (e.g. anointed as full professor just before retirement), due perhaps both to his impatient temperament and his free thinking, a combination that made him a charming and irritating^1^ intellectual *provocateur*.

On the one hand, he was the theoretical and critical voice who warned against the lure of market and policy demands that have strongly shaped scientometrics and have often caused harm via questionable evaluation practices, e.g. at the individual level:

…science policy makers in need of legitimation will probably have to be satisfied with ever more beautiful graphs from computers, claiming that these represent the structure and the dynamics of science. There exists a market and there is a technology available: as S&T students, we should know then what to expect! But I should like to caution researchers against the unreflective use of different statistical methods which sometimes — such as in the case of cluster analysis— provide results that are largely dependent on the choice of options offered by the computer programme. (…) This problem cannot be circumvented by ‘validation’ through interviews with experts (…). (Leydesdorff, 1987, pp. 320–321)

On the other hand, he was mainly ignored in the more sociological field of Science and Technology Studies (STS) (with few exceptions, such as Cambrosio) given that he conducted quantitative studies, as explained by Geoff Bowker (Bowker, 2020, p. 927):

I had a touching meeting with Loet Leydesdorff at the Social Studies of Science annual meeting about 10 years ago. He and I were saddened by the exclusion of scientometrics from social studies of science. (…) He pointed out rightly that scientometrics had been expelled from our rather shabby temple [of STS], and that even when they put on sessions, it was only those who already had the faith who attended. And the true paradox was that the qualitative and the quantitative had grown up together in science studies—they were richly intertwined, were exploring the same questions with different methodologies, were learning from each other. Then we witnessed the revenge of the quals. It didn’t matter that they hadn’t read, thought about, or heeded scientometrics—our emergent discipline was to be partly defined by the rigorous extirpation of statistical analysis. So much was lost along the way.

Leydesdorff was actually one of the very few researchers to attend regularly scientometrics, STS and innovation studies conferences, and to his credit, he always insisted in fostering bridges across various fields. For example, in 2020 he (together with Stasa Milosevic and myself) edited a special issue of the new journal *Quantitative Science Studies* with the aim of make it known beyond bibliometrics, with contributions from renown scholars from STS (Marres, Cambrosio), science policy (Hicks), economics of innovation (Frenken), institutional sociology (Heinze), scientometrics (Small) and library and information sciences (Borgman).^2^

## Loet Leydesdorff ‘s approach to science mapping

In the early 1980s, from his studies using various mapping techniques and setting a variety of parameters, Leydesdorff came to the conclusion that science mapping often generates results that are conditional on the choice of parameters among the options offered by the analytical technique, and that validations of science maps through interviews were not reliable because ‘experts tend either to condemn them out of hand or to rationalize the pictures presented to them’ (Leydesdorff, 1987, pp. 321). Therefore, he supported methodological pluralism, with the view that ‘[t]he multidimensional character of science is best captured by combining various perspectives, including disparate mapping techniques’ (Rafols et al., 2010, p. 1872).

### Journals as the main point of entry

**Fig. 1:**
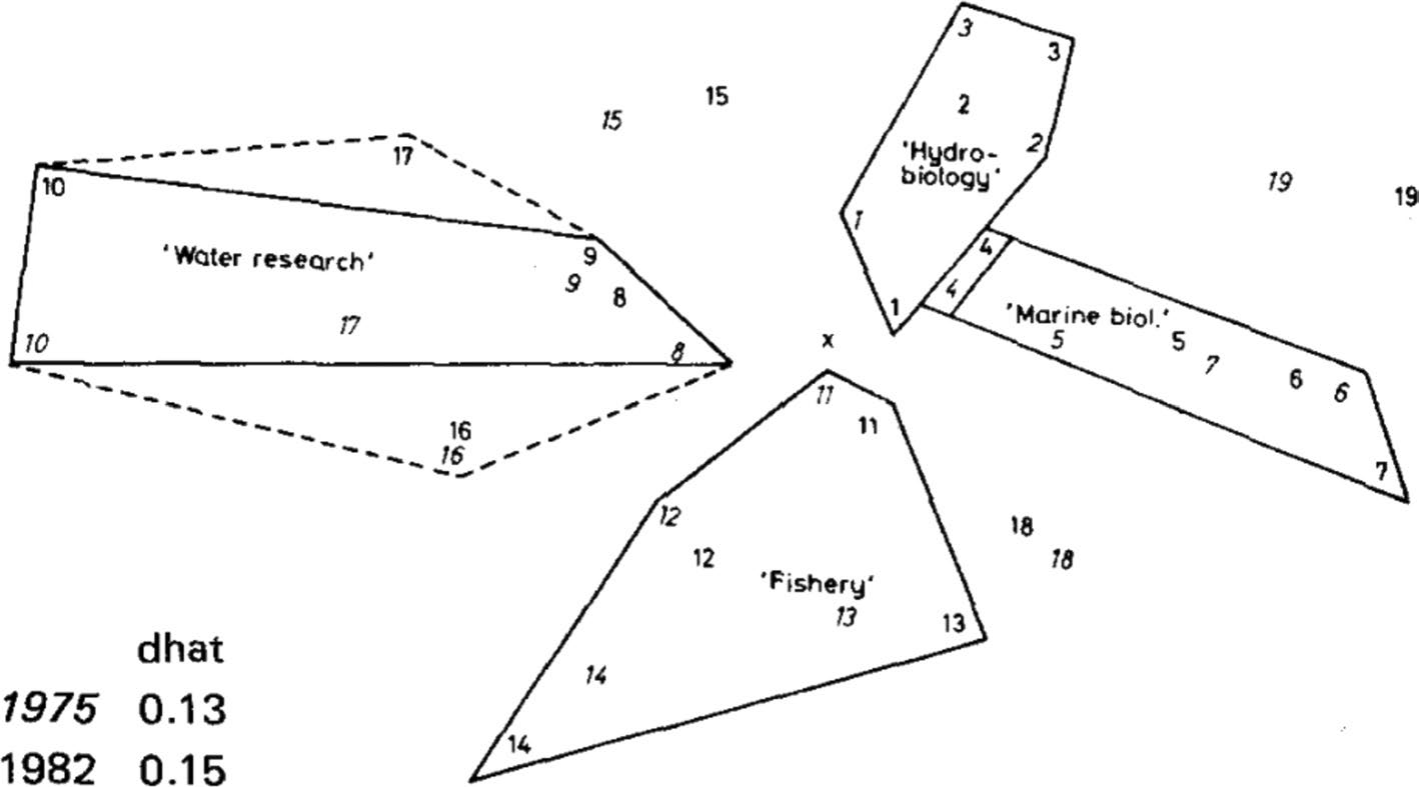
Epistemic distances among 19 ‘aquatic ecology’ journals in 1975 (journal number in *italics*) and 1982. Source: Leydesdorff (1986)

Whereas co-citation analysis was the preferred tool by Henry Small and colleagues working for policy at the Institute for Scientific Information (ISI)(H. Small, 1999; H. Small & Griffith, 1974), and co-word analysis was preferred by constructivists (Callon et al., 1986), Leydesdorff’s main approach was based on the use journal citation networks with data from the JCR, over which he would run factor analysis to reveal underlying cognitive patterns (Leydesdorff, 1986). Using citations as variable, ‘the factorial structure [of journals] is easy to interpret’ and ‘the designation of various factors [into scientific fields] does not give rise to serious problems’ (Leydesdorff, 1987, p. 314) as illustrated in Fig. 1.

With Susan Cozzens and Peter van den Besselaar, Leydesdorff applied this method to track scientific areas of strategic importance for policy, such as Artificial Intelligence (Leydesdorff & Cozzens, 1993; Leydesdorff et al., 1994). His preference for factor analysis remained a constant in his science mapping (Leydesdorff, 2006). Unlike clustering, which unambiguously assigns a category (e.g. a journal) to each cluster, the use of factor analysis shows which categories have loadings in various factors, which can be interpreted as a signal of interdisciplinarity. Indicators of interdisciplinarity was an issue of high interest to Leydesdorff that is closely associated with science mapping (Leydesdorff, 2007; Leydesdorff & Ivanova, 2021; Leydesdorff & Rafols, 2011a; Rafols et al., 2012). I will refrain from discussing interdisciplinarity in this article in spite our joint work since it requires a space of its own and in recent years we had arrived at divergent perspectives^3^ – though Leydesdorff might be upset that I avoid the chance to engage in discussion!

As explained above, the successful development of global maps of science based on clusters of journals (Boyack et al., 2005, 2009), triggered us to apply the methodologies that Leydesdorff had previously developed for journals at the level of WoS Categories, which are disciplinary aggregates of journals (Leydesdorff & Rafols, 2009).

**Fig. 2:**
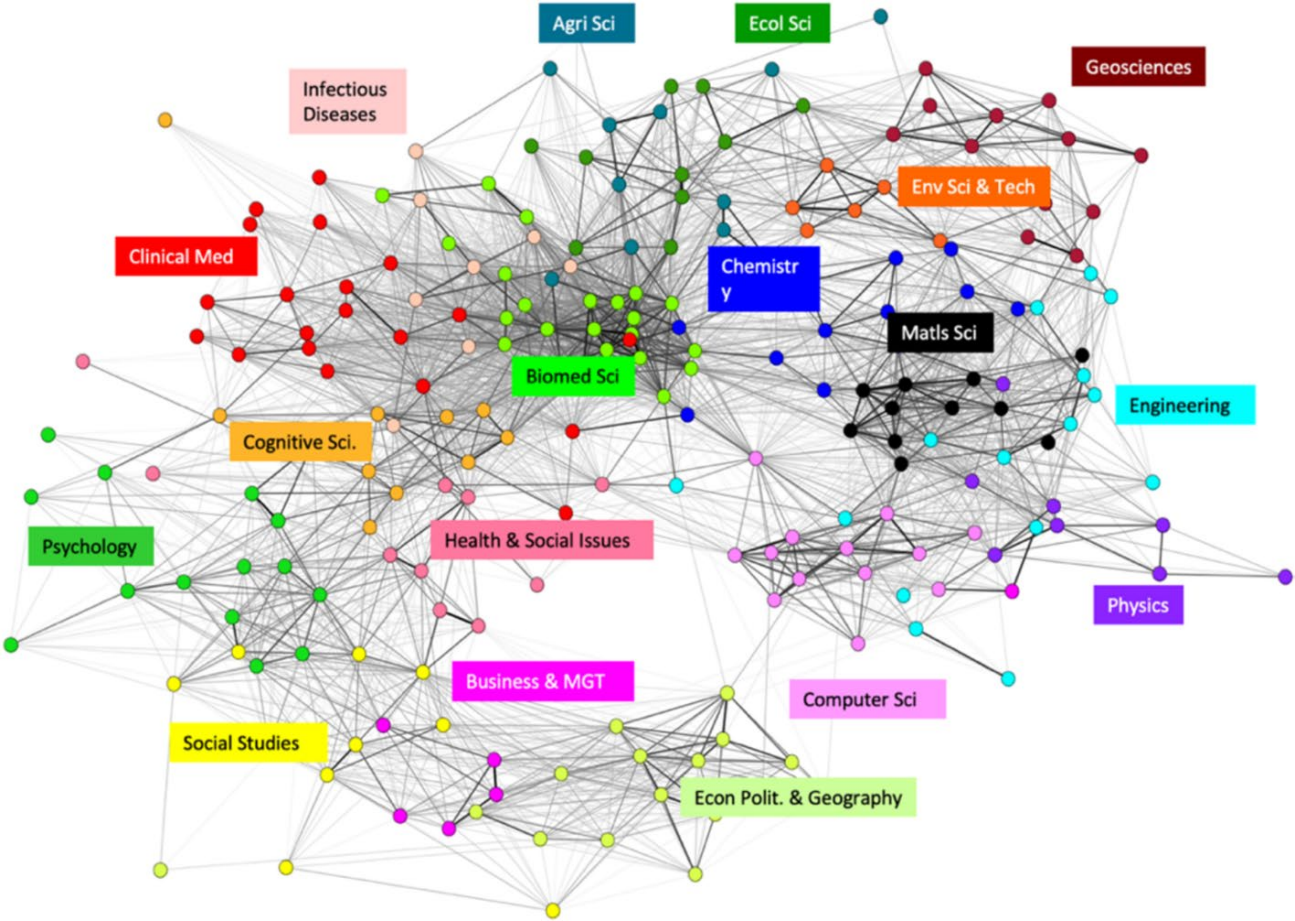
Global map of science based on the citation similarity across (disciplinary) Web of Science categories. Based on Rafols et al. (2010)

The approach produced a global map of science (see Fig. 2) that was surprisingly robust across various classifications, in spite of large degree of uncertainty and ambiguity in the assignment of journals to categories (Rafols & Leydesdorff, 2009). With the advance of computational capacity, it soon became possible to reproduce this map of science with a complete and interactive network of more than 10,000 journals, instead of aggregate disciplinary categories (Leydesdorff & Rafols, 2012a). Meanwhile, Klavans and Boyack (2009) showed through a comparison of twenty global science maps that the underlying structure of this map of science was also robust to changes in disciplinary classifications or clustering algorithms, even if some of the two-dimensional projections looked different. Currently, this type of mapping is publicly available at web sites such as https://sciencemap.eto.tech/.

Recent research has also noted the stability of the essential (simplified) structure of science, for example, in maps based on Wikipedia data for journal co-citations (Arroyo-Machado et al., 2020). Also according to topic modelling of publications (a technique that relies on text similarity of titles and abstracts), Hladík and Renisio (2025) confirm this stability and suggest, using principal component analyses, that the first axis of this structure is nature versus culture, and the second axis is life versus non-life.

**Fig. 3:**
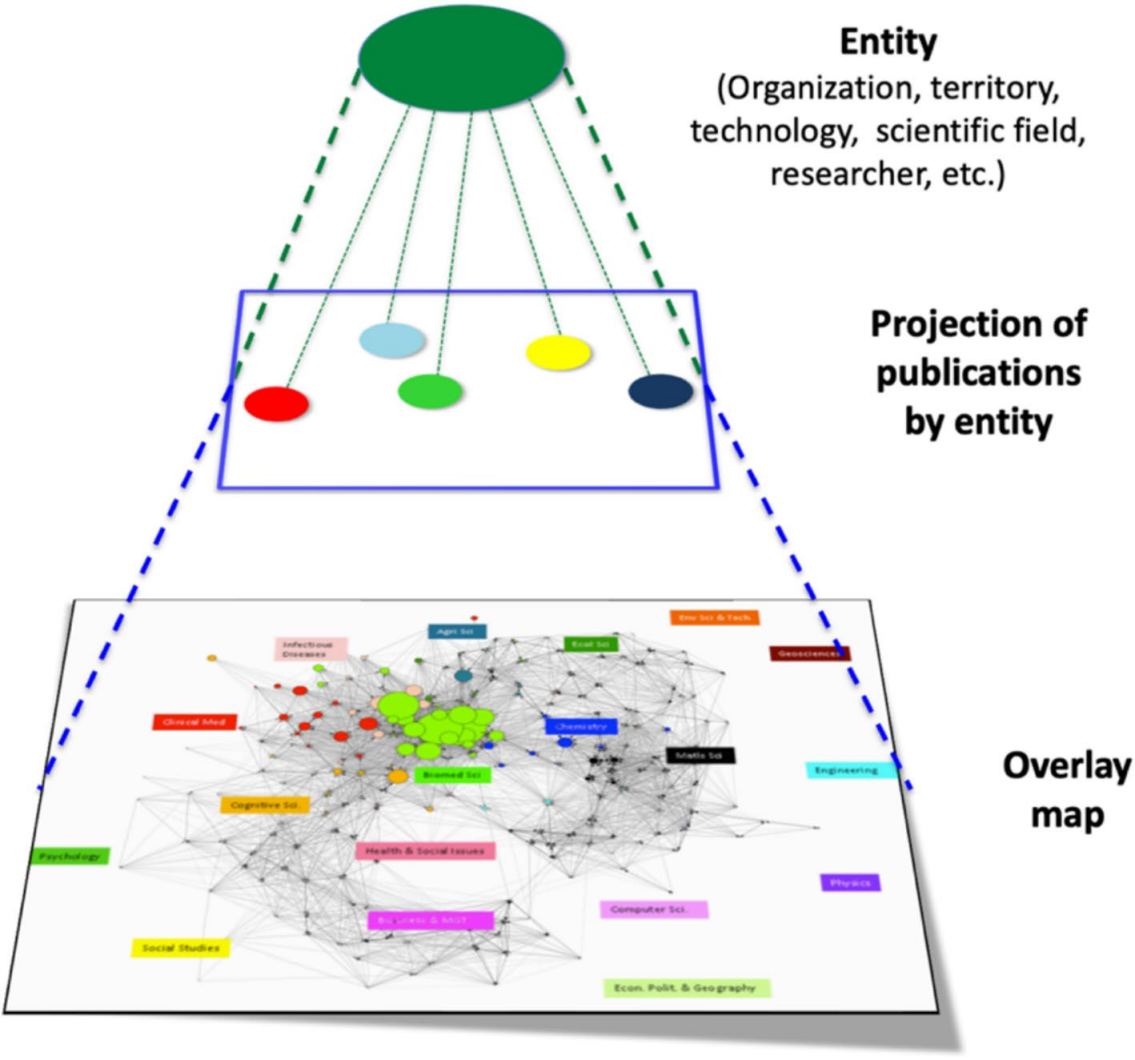
Overlay map, i.e. projection of the publications of an organisation (the European Molecular Biology Laboratory) visualising its position in the global map of science. Based on Rotolo et al. (2017)

The fact that the overall structure of science was found to be quite robust to changes in classifications or clustering algorithms, allowed to think of maps of science similarly to the way we think of a geographical world map: as a reference or a basemap upon which we can project (overlay) the bodies of knowledge associated with a certain cognitive, institutional, organizational or geographical entity (Rafols et al., 2010), as illustrated in Fig. 3. Unlike representations of categories in lists or bar-charts, these maps captured the epistemic positions and distances between the objects analysed (e.g. organisations) over a shared frame of reference. This additional piece of information, the epistemic distance (also called proximity (Frenken, 2020) or relatedness (Balland et al., 2019) in economic geography or disparity (Stirling, 2007)) is relevant in evolutionary theories of innovation: since science and innovation mostly evolve through explorations in neighbouring spaces, the scientific trajectories (and associated innovation pathways) can be described as the paths walked over epistemic spaces (Rotolo et al., 2017; Stirling, 2024). Therefore, keeping the global map as a fixed frame of reference was important because it made it possible to analyse research dynamics over an epistemic space. This is useful in evolutionary theorizing, and it also allows benchmarking for exploratory policy analysis, e.g. in emergent technologies.

Leydesdorff made it possible for other labs to re-use this global (referential) map of science by sharing all the data, information and scripts in his website,^4^ in a then unusual display of Open Science *avant la lettre*, which he applied to most of his work. This allowed popularise the notion of overlay, i.e. the idea of keeping the cognitive structure of the global map of science fixed and projecting a body of knowledge over it (Rafols et al., 2010).

### Cognitive vs. social representations and centrality of dynamics

The method of overlay mapping has some theoretical implications. On the one hand, the stability of the global basemap (the ‘backbone of science’), facilitates comparison across organisations, time or scientific developments. On the other hand, in agreement with Leydesdorff’s tenets, it allows visualise the co-evolution over the epistemic spaces of analytical objects that represented either science, market and government, as explicitly stated in the Triple Helix metaphor (Etzkowitz & Leydesdorff, 2000). While Leydesdorff was concerned with the interactions of science with social and political arenas, he thought that it was better for analytical clarity to separate the epistemic and social dimensions: on the one hand, the social organisation, and on the one hand (and his core interest), the cognitive structures derived from scientific communication practices.

This conclusion is at the root of the Amsterdam Science Dynamics programme: the analysis of scientific contents and the analysis of research institutes must be pursued as related but separate efforts. Science dynamics is then essentially the multi-level problem of the combination of insights gained from these two complementary forms of analysis.”(Leydesdorff, 1986, p. 104)

We used this approach of separate but related analytical dimensions in order to describe developments in emergent technologies over cognitive and geographical spaces (Leydesdorff & Rafols, 2011b; Rotolo et al., 2017). This approach, based on the social versus cognitive duality, was in contrast to the heterogenous networks (with researchers, cognitive categories and non-human actors in the same network) used by researchers following Actor Network Theory such as Callon and colleagues (Bourret et al., 2006). However, Leydesdorff’s analyses were always very attentive to the dynamics of science as shaped by social forces. He didn’t conceive science maps as fixed structures, but as structures that evolved both by themselves and in interaction with the social world, in agreement with STS scholars but in contrast to the more positivistic and Mertonian scientometricians.

Our mapping efforts also included the creation of a global patent map based on the International Patent Classification (IPC) at 3 and 4 digit level (Leydesdorff et al., 2014). However, unlike the global map based of science based on publications, the structure obtained was difficult to interpret, as happened to parallel efforts (Kay et al., 2014; Schoen et al., 2012). One difficulty was the multiplicity of logics of the categories of patent classification: the same patent (e.g. salad bowl with cooling function) could be classified in multiple classes with different logics (e.g. adaptation to climate change, refrigerators, storehouses, food preservations), an issue we will discuss later. Table 1 summarises Leydesdorff's contributions to science mapping according to various perspectives and methods, mainly related to different choices with regards to the ontologies used.

**Table 1:** Specifications of sensors used in the experiment

**Contributions**	**References**
Use of journal-to-journal citation data	(Leydesdorff, 1986)
Pore water pressure sensor	(Leydesdorff, 1987)
Mapping of emergent scientific fields with journals	(Leydesdorff, 1994; Leydesdorff et al., 1994; Van Den Besselaar & Leydesdorff, 1996)
Classification of science according to factor analysis of journal citations	(Leydesdorff, 2006)
Global map of science based on Web of Science Categories.	(Leydesdorff & Rafols, 2009; Rafols & Leydesdorff, 2009). Updated in (Leydesdorff, Carley, et al., 2013; Carley et al., 2017).
Overlays maps of science	(Rafols et al., 2010, 2012)
Maps on the diffusion of emergent technologies	(Leydesdorff & Rafols, 2011b; Rotolo et al., 2017)
Global maps based on journals	(Leydesdorff & Rafols, 2012b; Leydesdorff, Rafols, et al., 2013; Leydesdorff et al., 2016)
Global maps of patents	(Leydesdorff et al., 2014)
Global maps of Medical Subject Headings (MeSH)	(Leydesdorff et al., 2012; Petersen et al., 2016)

### Sociological theorizing

In contrast to most scientometrics, Leydesdorff developed his mapping efforts in dialogue with sociological theorizing, i.e. generating sociological insights about science dynamics from the patterns observed.

“In recent years, an awareness has emerged that serious attempts should be made to connect the more qualitative kind of sociological theorizing, with its increasing focus on the cognitive aspects of science, with the more quantitative approach of scientometrics, characterized by its increasing awareness of the relevance of institutional factors. This (…) implies a model of science not only at its various internal levels, but also in relation to important external factors (such as funding). The development of this type of theorizing is a long-term academic goal, as opposed to the short-term need for science policy indicators in the political arena.” (Leydesdorff, 1987, p. 320)

For example, the hierarchical classification of Medical Subject Headings (MeSH) of MEDLINE made it possible to map distinct conceptual dimensions by means of three branches of the hierarchy: branch C for Diseases; D for Chemicals and Drugs, E for Analytical, Diagnostic and Therapeutic Techniques and Equipment. (Leydesdorff et al., 2012). This mapping approach has later been used for example to show the degree of cognitive proximity between the research of diseases listed in grants and the diseases of the resulting papers (Coburn et al., 2024). Leydesdorff and colleagues later proposed to interpret these three dimensions in terms of demand, supply, and technological capabilities, respectively, and analysed synergies between the three dimensions in terms of a ‘triple helix’ (Petersen et al., 2016).

Another example of this sociological theorizing over science mapping can be found in Leydesdorff’s interpretation of the patterns of diffusion of two emergent technologies, small interference RNA (siRNA) and nanocrystalline solar cells (NCSC), which he proposed to relate to disciplinary-oriented (“Mode 1”) to transfer-oriented (“Mode 2”) research, respectively. I have to confess that I didn’t find these ex-post insights, based only on the evidence from bibliometric data, to be sufficiently robust. Yet, I still think that it is valuable to go beyond the purely descriptive and try to gain sociological insights. I believe that more qualitative analysis such as interviews is needed to interpret the patterns observed in the maps – this was a point of friction in our collaborations. To this day, I see a weakness in the relative lack of mixed-methods in STS and sociology of science (especially painful in my own research!), with few exceptions (Bone et al., 2020; Held & Velden, 2022).

## Current challenges in science mapping

**Fig. 4:**
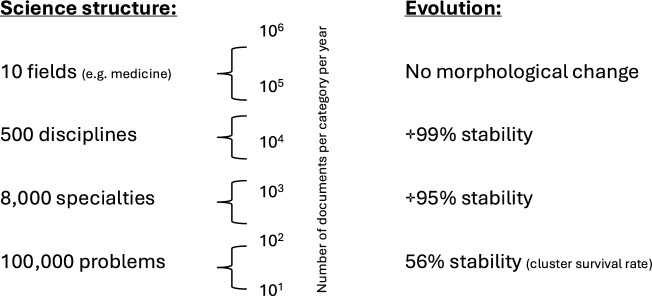
Levels of aggregation at which the structure and evolution of science can be analysed, according to the conventional perception of science as nested epistemic categories. Evidence suggests that there are different (and non-coherent) categorisation logics as we move to lower aggregation. Based on Boyack et al., (2014a, 2014b)

The maps that Loet Leydesdorff and his collaborators developed were at a high level of aggregation, either at the journal or groupings of journals (such as WoS Categories) and thus they described rough disciplinary patterns. However, many journals are well known to publish a substantial share of articles that don’t fit with their disciplinary scope. Therefore, the consensus among bibliometricians around 2010 was that more fine-grained maps were needed. The expectation was that bottom-up techniques such as article-level clustering would provide a “more accurate taxonomy” (as worded by Klavans and Boyack (2017)) of the underlying structure of science. The assumption was that science had a hierarchical cognitive structure, one of nested categories at four main levels, with some 10–30 fields, 200–500 disciplines, about 8000 research specialties and 100,000 problems, as described in Fig. 4.

In the last 15 years, improvements in computational power and clustering algorithms have allowed the creation of large maps of science based on article-level clustering built with 10 to 40 million documents, 5 to 500 million citations, and 4000 to 100,000 clusters (see Table 8.2 in Boyack & Klavans, 2019, p. 198). Boyack and Klavans, of the consultancy SciTech Strategies, pioneered the technique of citation-based article-level clustering which eventually became Elsevier’s’ product SciVal, and thus has become an application for research management. They have tested the clustering with cards that describe each cluster in terms of keywords, journals and main authors. According to Boyack and Klavans, researchers can quickly identify their cluster and neighbouring ones (Klavans et al., 2012). However, this validation has not been fully described nor published, nor I am not aware of independent researchers being able to test it. Topic modelling is another epistemic classification method, in this case text-based, that faces a similar challenge of understanding and ‘validating’ the topics algorithmically generated in a bottom up manner (Cassi et al., 2017; Hladík & Renisio, 2025).

Making sense of specific clusters or topics is sometimes very easy and sometimes rather confusing, with many fuzzy boundaries. As mentioned above, Leydesdorff was sceptical of the robustness of validation methods (Leydesdorff, 1987, pp. 321). In an early debate on the robustness of co-citation clusters, Diana Hicks also warned that validation based on researchers’ readings of clusters of topics (or specialties) is problematic because the complexity of the information provided may have a generalization effect, making it possible for ‘for a scientist [who is not a bibliometric expert] to find support for existing conceptions of the specialty’. This phenomenon, she argues, has been shown to happen in people’s agreement with astrological ‘descriptions couched in generalized terms [which] allow “the individual to perceive and interpret the information in a many which is congruent with the existing self-concept’ (Hicks, 1988, p. 382).

Waltman and van Eck at CWTS developed an approach similar to SciVal to produce clusters based on single citation clustering of documents for the database WoS (Waltman & van Eck, 2012). A related clustering algorithm has recently been implemented for the database OpenAlex and used to generate overlay maps (Haunschild & Bornmann, 2024; Van Eck & Waltman, 2024). Given that the main purpose of this clustering is the citation normalisation for the Leiden Ranking,^5^ it is conducted with about 4000 clusters, in comparison to the 50,000–150,000 clusters often used by Klavans and Boyack and others in efforts more focused on topic description. This raises that question of the level of aggregation at which clusters of paper are useful to capture research problems.

Pragmatically, here I focus on the CWTS classification since it is fully open and researchers have been able to scrutinise it, with mixed results. Haunschild et al. (2018) looked at CWTS clusters related to chemistry and found that they were highly spread across many INSPEC and WoS (i.e. subdisciplinary) categories. Bascur et al. (2024) have analysed the agreement of CWTS cluster with MeSH and also found that the degree of overlap is very variable depending on topics. The agreement is highest for diseases and organisms, and lowest for disciplinary or professional categories, irrespective of whether the clustering is based on citation or semantic similarity. In the next two subsections, let us examine in detail the challenges that pose algorithmically generated bottom-up classifications.

### Mapping invasion biology

To my knowledge the most detailed and insightful analysis on the contents of the article-based citation clusters is the one carried by Held and Velden (2022). Held and Velden (2022) conduct a mix-methods case study on the field of ‘invasive biology’, comparing different citation clusters, building on two previous critiques (Held, 2022; Held et al., 2021).

**Fig. 5:**
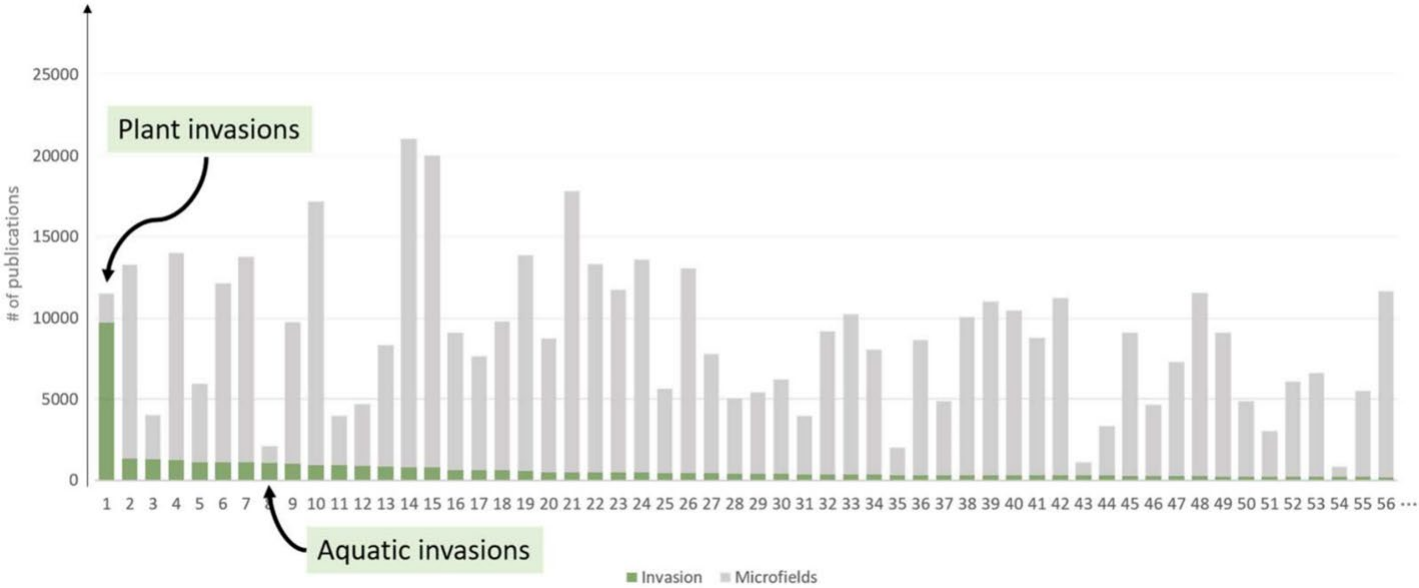
Distribution of ‘invasion biology’ publications (in green) as found by keyword search across 56 CWTS citation clusters with the highest number of publications (with number of papers per cluster in grey). The two cluster with more than 40% of publication in ‘invasion biology’ are highlighted. Source: Held and Velden (2022)

In this excellent study, Held and Velden review the approaches to citation-based clusters against theories of citation (including (Amsterdamska & Leydesdorff, 1989)). Following Seitz et al. (2021), they propose that the epistemic dimension of citations links are indicative of a different epistemic dimensions of research: the research problem addressed, the empirical object studied, the methods used, the theoretical resources used, or the external relevancy of the study and its results. I would highlight two findings:

- Publications on ‘invasive biology’ according to search keywords are spread over many clusters as shown in Fig. 5. However, in only two clusters (highlighted in green) ‘invasion biology’ makes more than 40% of the publications.
- The contents of the cluster correspond mainly to empirical objects of study: either groups of species (invasive plants, crustacea, seaweed, ladybird, amphibians, small mammals, etc.) or contexts (ballast water, aquatic plants in wetland and lakes). There are some clusters that correspond to theoretical problems (pollination, species distribution modelling, hybridization and genetic structure).

Following these observations, they state that:

Obviously, the ordering of publications by empirical object of research, which the direct citation network delivers, is only one of potentially many useful thematic perspectives on the published knowledge base in invasion biology. An alternative thematic perspective that focuses on methods might distinguish field-observational studies, experimental studies, macroecological studies, and policy-oriented social studies. Yet another thematic perspective might foreground the theoretical understanding of invasion processes and distinguish work by conceptual focus: from invasion pathways and factors of invasion success, to forms of invasion impact. In principle, by use of different approaches and types of data one should be able to tease out different aspects of the topic structures in a research specialty. (Held & Velden, 2022, p. 666)

And they conclude that clustering based on citation relations ‘results in a blurred signal’ and that therefore ‘global science maps are likely not adequate to capture all specialties.’ (Held & Velden, 2022, p. 669).

Nevertheless, the two science maps they generate (one by local clustering and the other from CWTS global clusters) show a similar structure in terms of the relations between empirical objects: marine invasion related to freshwater invasion, freshwater invasion to invasive terrestrial vertebrates, to invasive plants, to three clusters of invasive insects. Thus, although the categories may differ, the relationships among the categories are still quite similar. At the level of disciplinary categories we had also observed structural consistency in spite of large differences in the distributions of paper or journals across categories (Rafols & Leydesdorff, 2009). This similarity can be explained the same way that impressionist paintings manage to create images without precision in the brush strokes.

### Mapping mental health

In a recent study on mental health for the Swedish funding agency Vinnova,^6^ we used CWTS clusters to describe the research structure of this issue, aiming at visualising the relative research efforts across different approaches, for example in terms of biological mechanisms, social determinants, prevention, therapeutics, public health interventions (Van De Klippe et al., 2023). In agreement with Held and colleagues, we also found that the content of the clusters was focused on empirical objects, but in our case, the additional challenge was that the empirical objects responded to multiple and incommensurable logics (mental disorders, but also social determinants, materials, healthcare interventions).

**Fig. 6:**
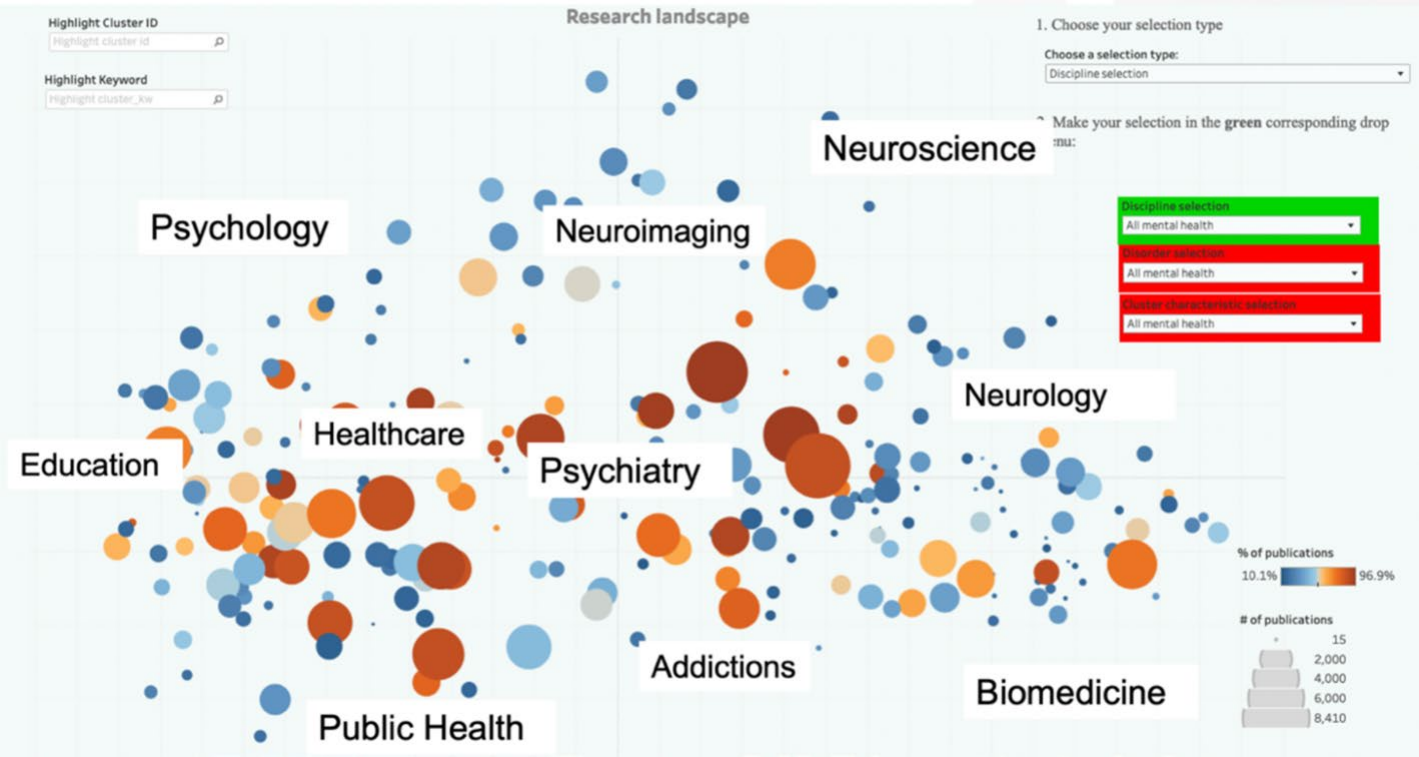
Map of 380 CWTS clusters related to mental health positioned according to citation similarities. Size of nodes indicates number of publications related to mental health in each cluster. Brown colour indicates high % mental health in each cluster; blue colours indicate low % of mental health. Based on Van De Klippe et al. (2023). Interactive web site at: https://public.tableau.com/app/profile/tim5920/viz/MentalHealthtool/MHtool

We selected 380 cluster (out of 4013 clusters) that had at least 10% of publications related to mental health according to MeSH descriptors. We placed these clusters on a map of science according to their citation similarities and obtained a plausible epistemic structure that allows some sense making (see Fig. 6): a triangular space with topics related to psychology in the left side (from social science in the bottom left to experimental psychology in the top), with neurosciences in the right side (from experimental brain research in the top vertex to biomedicine in the bottom-right), and with health disciplines in the bottom (from biomedical application in the bottom-right to public health in the bottom left).

In this study, we also encounter some of the main challenges reported in the studies by Held. First, the long tails in distribution: even if we include all clusters (380) with more than 10% of publications related to mental health, about 20% (!!) of the corpus remains spread over the other 3633 clusters. Second, WoS categories are not associated with a few (10 or 20) core disciplinary clusters as would be expected in a nested structure; instead within each cluster there are many WoS categories; this corroborates the insights that clusters don’t follow a disciplinary logic. Third, the topics of clusters are sometimes cohesive and thus easy to label (e.g. autism), but sometimes difficult to interpret (e.g. cognitive control and electroencephalography). Fourth, the logics of the topics of clusters are conceptually very different. This occurs not only in terms of methods, theory and epistemic objects as described by (Held & Velden, 2022), but more worryingly for our analysis, the logics for epistemic objects differ. Clusters show logics related to focus on:

- *Mental disorders*: e.g. autism, attention deficit hyperactivity disorder, bipolar disorder, schizophrenia.
- *Social determinants*: e.g. school bullying, stress and burnout syndrome, income inequality, homelessness, food insecurity
- *Healthcare interventions*: e.g. palliative care, telemedicine, community treatment, psychoanalysis, mobile health
- *Research materials*: e.g. amyloid precursor protein, ketamine, cerebellum, D-amino acid oxidase.
- *Methods*: e.g. functional Magnetic Resonance Imaging (fMRI), brain-computer interface**.**

In some cases, these distinctions were useful for the purpose of the project to distinguish between different approaches for a same condition. For example, in the case of dementias, we found a large cluster focused on psychiatric diagnosis, one on biochemistry (amyloid precursor protein), and another on caregiving. However, in the case of bipolar disorder most publications are in one single cluster dominated by psychiatry, while in the case of major depression, the publications are spread across clusters related to healthcare, treatment, experimental psychology, brain mapping, transcranial stimulation and pharmacology.

In summary, the logics of contents behind these clusters is unpredictable and apparently capricious, with only some methods, some research materials, some disorders, and some socials determinants being depicted. As explained by Held & Velden, 2022 (p. 655) “…only some topics or specialties are captured by a given map, while others are left out. As of now, we cannot specify under what circumstances which types of topics or specialties are reconstructed.”

These observations suggest that certain uses of the clusters for science policy and management are problematic (although they are actually being used currently in the most popular databases, OpenAlex, WoS and Scopus!). We face two challenges here. First that there is a multiplicity of ontologies of topics (various parallel logics for classification), and second that each publication can belong at the same time to categories of the various ontologies. For example a paper can be about fMRI (method), Alzheimer’s disease (disorder), and caregivers (healthcare intervention). Some Natural Language Processing (NLP) methods such as topic modelling, overcome the second challenge by assigning a paper to multiple categories but they still face the problem of producing multiple classification logics. For example in the analysis of obesity research, we found topics of specific conditions related to obesity such as diabetes, hypertension or cholesterol (Cassi et al., 2017).

**Fig. 7:**
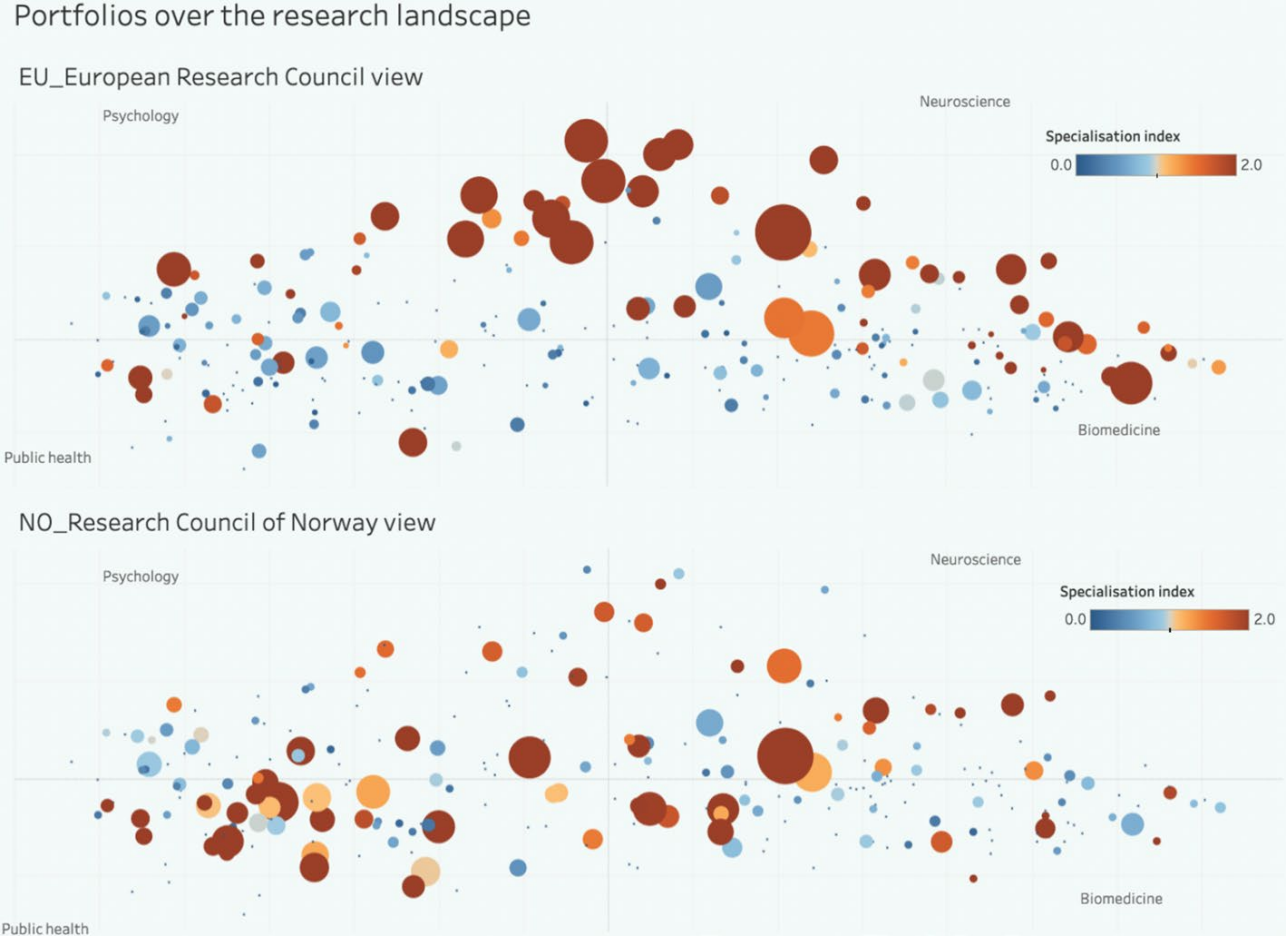
Overlay of the publications (2015–18) of the European Research Council (ERC) (top) and the Research Council of Norway (RCN) (bottom), on clusters related to mental health. See Fig. 6 above to locate disciplines. Brown colour indicates high level of specialisation in that topic. The ERC specialised on brain mapping and biomedical neurosciences, while the RCN had a more diverse portfolio, with some focus on public health and healthcare interventions. Based on Van De Klippe et al. (2023). Interactive website at: https://public.tableau.com/app/profile/tim5920/viz/MentalHealthtool/MHtool

In spite of these logical inconsistencies, the science map for mental health was useful as a heuristic to locate whether a given organisation, funder or disorder is more or less related to biomedical, psychiatric, brain mapping, healthcare interventions or public health approaches. It made it possible to compare specialisation in topics across countries and to visualise stark contrasts in funders portfolios. For example, ERC funding was very tilted towards brain mapping, the Swedish health council towards public health and the Spanish health council towards biomedicine, as illustrated in Fig. 7.

## From maps to mappings: toward multiple ontologies

### Co-existence of multiple ontologies and heterarchy

In his essay *The Analytical Language of John Wilkins,* Jorge Luis Borges proposes a shockingly incongruous classification of animals (Borges, 1942): all classifications are inevitably arbitrary and contextual, based on idiosyncratic interests and assumptions situated in a certain place and time. The quest for an objective and ideal classification is a fantasy, a chimera. Foucault built on this idea to explore how different historical periods use different ways of thinking about truth and about discourse – what he called *epistemes* (Foucault, 2012).

A key insight from the case studies of mapping reviewed above is that there is not a natural structure of science. These cases contradict the common vision of an essential structure of science based on nested epistemic categories, in which the most fine-grained categories would be research problems that could be grouped into research specialties that could be grouped with disciplines that could be grouped into disciplinary fields as illustrated in Fig. 4 (Boyack et al., 2014a, 2014b). Both in article-level classifications and topic modelling, we have found that most scientific problems or specialities cannot be assigned to a single academic discipline. Instead, research problems and specialties overlap and can be constituted and classified with a variety of contrasting ontologies (e.g. methods, theories, materials). In the case studies above that citation-based clusters tend to be constituted around empirical objects (or constructs) but even then, the logics of these objects can be disparate: research materials (beta-amyloid protein), mental disorders (autism) or social determinants (bulling or poverty) as shown by the case of mental health.

The expectations that article-level clustering or topic modelling would produce more “accurate” and robust science mapping have not been fulfilled. As Borges and Foucault, we have now seen that these classifications can be very fine-grained, but they contain categories belonging to multiple logics. Instead of a single and hierarchical epistemic structure, scientific categories form heterarchical networks.

This is not a surprise for anthropologists and historians of science. In *The body multiple,* an ethnography of atherosclerosis, Annemarie Mol describes how the medical practice of this disease involves multiple medical practices and understandings depending on the times and contexts. In the different diagnoses, treatments, medical specialties and patient experiences, atherosclerosis becomes a different thing as it travels across different objects, discussions, observations and measurements. What might be expected to be a single and unique entity (atherosclerosis) from an ontology focused on disease, is actually a variety of entities in multiple ontologies that ‘inform and are informed by our bodies, the organization of our health care systems, the rhythms and pains of our diseases, and the shape of our technologies.’ (Mol, 2002, p. 7).

Peter Galison also observes that instrumentation, experimentation and theory are not closely knitted to each other and that each of them evolves with intercalated breakthroughs (e.g. in quantum physics the same instruments and experiments were used before and after the theoretical revolutions of the 1920s) (Galison, 1999). Therefore, we should not expect methodological bodies of knowledge to be homogenously classified with theoretical bodies of knowledge. Instead a same method can overlap with various objects of experimentation and with various theoretical bodies.

### The choice of relevant epistemic spaces and projections

The implication of acknowledging multiple ontologies is that the analyst can decide which dimensions of science she wants to make visible by choosing to map through particular characteristics of some specific units of analysis (e.g. publications, grants, news). The key questions then are about which logics, characteristics and relations are relevant for particular analyses. The answer to these questions will determine which ontologies and classification to use, which epistemic spaces will be generated and thus the properties and relations that will be made visible.

**Fig. 8:**
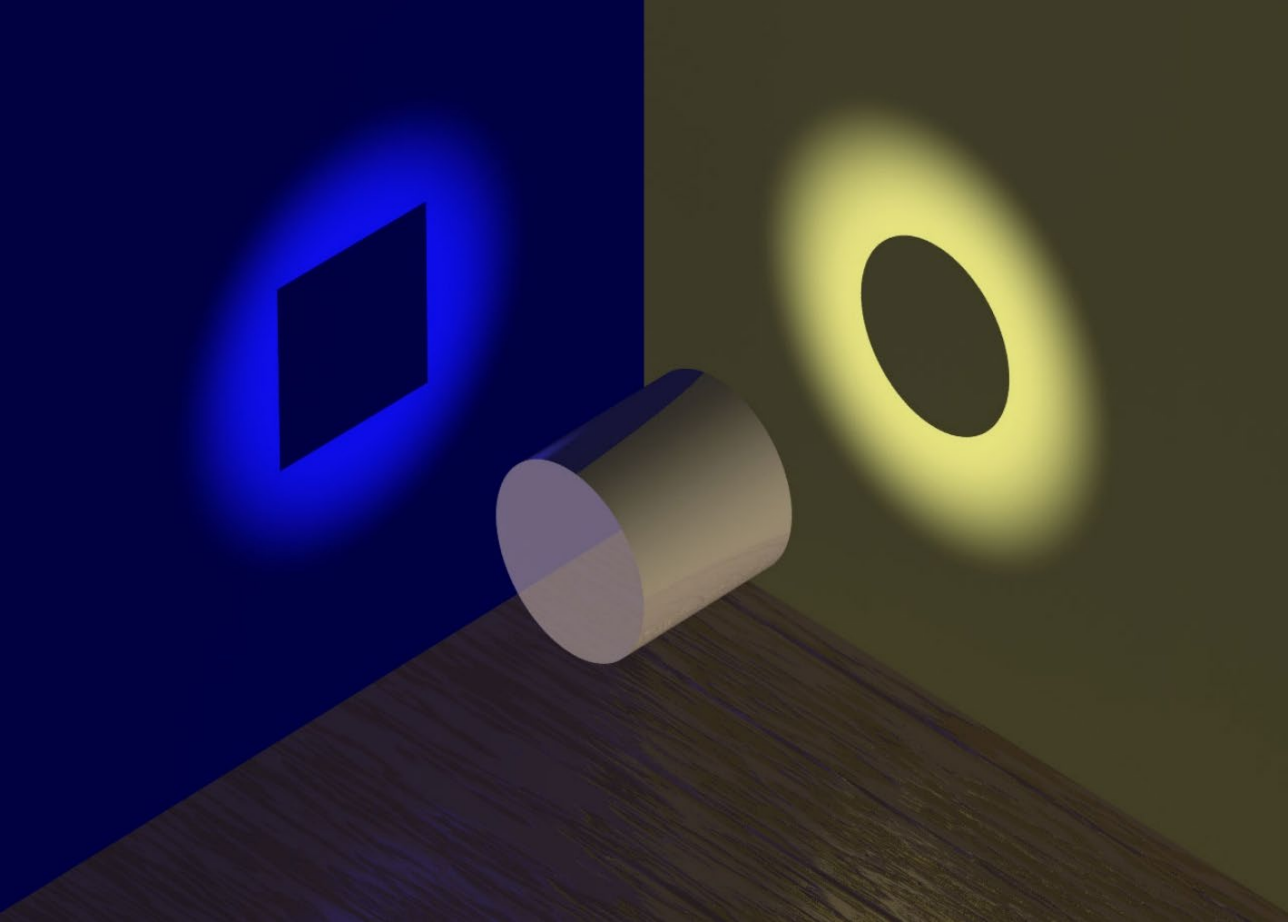
The projection of shadows of a cylinder on two perpendicular walls illustrates how the changes in perspective can produce very different representations when dimensions are reduced.

The representation of an epistemic space in a map is a reduction from a highly multidimensional space to a two-dimensional space. Therefore, many different projections are possible. This is illustrated in Fig. 8 by how a change in the angle of projection of a simple object such as a cylinder, can transform the shadow of the same cylinder from a blue square into a yellow circle. If this happen with a reduction from two to three dimensions of an object with such a simple geometry, just imagine the many possibilities of projection that can be produced from highly-dimensional object into the shadows of a two-dimensional wall by changing the angle of the projection – yet all of these are ‘truthful’ projections of the same object!

The choice of angle of projection is not neutral or innocent. Choices of classification (Bowker and Star (2000)) and ontology (Mol (2002)) can have major social consequences, may result in conflict and may need to be settled by political negotiations. Which is why institutions play such a central role in classifications. The anthropologist Mary Douglas (1986, pp. 91–109) nicely explains how ‘institutions do the classifying’ through the examples of the transformations in wine and jobs classifications. She describes the extraordinary changes that the classification of jobs in the *Dictionnaire Universel du Commerce* underwent from the late 18th, when guilds were the dominant institution, to mid nineteenth century, under the influence of free trade institutions after the industrial revolution.^7^ Douglas also explains how wine classification shifted from territory-based classification in France to grape types in California (i.e. *terroir* vs. *cépage)*, after the internationalisation of wine markets in the late twentieth century meant that buyers lost the detailed territorial context.

These radical changes of perspective remind us not only that our science maps reflect the structure of science under a given perspective rather than an essential pattern. In fact, rather than a ‘map of science’, we could talk of ‘mappings of science’. Here, I would emphasize that the gerund ‘mapping’ expresses the process of the projection of complex sociotechnical spaces to a simplified epistemic space, which is conditional on the descriptive variables selected. The simplification is useful because it helps us to visualise more clearly some patterns at the expense of hiding others, by choosing the classification and mappings that respond to particular analytical needs. Thus, the selected mappings are not those which are more “accurate”, but those which prove more useful for answering our questions.

Following Douglas, Bowker and Star, we can think how the choices of ontologies and classifications are shaped by the dominant institutions in science and their transformations. One may wonder if the conventional assumption that the (relevant) bodies of knowledges are disciplines and that they are self-evident, is related to the mainstream of academies and universities in terms of disciplines. In contrast, meso-level research dynamics is strongly shaped by scientific specialties that may have different logics, such as materials (for example Small’s seminal work illuminated collagen research (H. G. Small, 1977)). In mental health, we have found dementia clusters related to research materials (amyloid precursor protein), others to medicine (diagnosis of Alzheimer) and other to healthcare (caregivers in dementia). Even if all these clusters are related to dementia, the logics of the second (diagnosis) and third (care) clusters show a shift towards a classification that makes visible social demands on science.

Hence, not only there is no structure of science, but the boundary between science-driven and socially-driven topics (and their classificatory logics) is fuzzy and ambiguous (Knorr-Cetina, 1982). Now, if science is undergoing a major transformation or at least broadening its remit towards more socially-driven and mission-oriented modes of research, this should have serious implications for the types of classifications and ontologies used in science mapping (Gibbons, 1999; Mazzucato, 2018; Schot & Steinmueller, 2018).

An example of a change in classification driven by policy needs is the quick adoption by database providers and consultancies of tags for publications related to Sustainable Development Goals (SDGs). In this case, there is little consensus on tagging: agreement in assignments of papers to SDGs across databases is in the range of 25% to 40% (Armitage et al., 2020; Purnell, 2022). Moreover, in these classification relatedness to SDGs does not distinguish types of problems (e.g. whether disease of the rich or neglected diseases, all contribute to health) or descriptive versus action-oriented and transformative research (Rafols et al., 2022). In terms of politics of classifications, one can speculate that for SDG-labelling, dominant institutions in science (commercial providers and traditional universities) are using the SDGs labelling for legitimation without engaging in a major transformation of their research agendas.

Lack of engagement with the politics of classification and ontologies is likely to support the *status quo*. An interesting perspective on the role of critical views through statistics is provided by Boltanski (2014). Asking ‘Which statistics for which critique?’, Boltanski responds that one can differentiate between reformist critique, which would use existing statistical instruments (as Bourdieu’s analyses of French education according to social class (Bourdieu & Passeron, 1964)), and radical critique, which has more difficulties in using existing statistical approaches since its needs new observations. “In the latter case, the statistical enterprise must take charge of both the numerical counting operation and the formulation of the categories on which the claim to modify the bases of social accounting necessarily rests." (Boltanski, 2014).

Boltanski’s position, translated to the context of bibliometrics, would say that the mobilisation of existing ontologies, classifications and mapping techniques can help in reformist critiques. However, radical critiques would need new ontologies or classifications. In this sense, bottom-up mappings can be useful for pointing out the need for new ontologies when they produce evidence of lack of fit between empirical patterns and conventional categories.

For example, in health research policy, a reformist critique would be the comparison of research efforts against disease burden in middle income countries, showing that they should rebalance their health research portfolio to address the diseases with higher burden in their countries (Kumar et al., 2024). For this approach there exist large institutional efforts to collect data on publications and disease burden estimates. Transparency and openness of the data is then important to allow users to scrutinize the underlying data and classification choices (as suggested by the Barcelona Declaration on Open Research Information: https://barcelona-declaration.org/). However, a more radical critique would say that thinking in terms of diseases is not enough, that studies on how to improve health systems and increase overall research capacity is also important (Coburn et al., 2023) – but doing this type of analyses requires a different ontology and categorisation system, which have not yet been developed.

But to which extent can these discussions on classifications and ontologies be operationalised in practice? Let us now examine how we could move in this direction, following the heuristics proposed by Andy Stirling for opening up policy appraisal (Leach et al., 2010; Rafols & Stirling, 2021).

### Mapping science with multiple ontologies

Many studies have made comparisons across different types of bibliometric classifications, generally disciplinary (Rafols & Leydesdorff, 2009), but also with citation clusters (Haunschild et al., 2018; Klavans & Boyack, 2017) or with MeSH categories (Bascur et al., 2024). There is also some work in mapping in parallel cognitive and social dimensions (generally collaborations), but there is much less mapping that uses multiple analytical dimensions, including e.g. geographical, technological or institutional aspects (Bone et al., 2020; Rafols, 2014), following the multidimensional notion of proximity developed in economic geography (Boschma, 2005). One of Leydesdorff’s contributions in the last ten years was to analyse emergent technologies with these multidimensional lenses (Rotolo et al., 2017).

However, the challenge identified in Sect. "Current challenges in science mapping" with current science maps is perhaps more subtle, since in principle we are dealing with the same analytical space (an epistemic space), but observed with lenses that are mix various types of ontologies. This problem in algorithmic science mapping is the same encountered in patent classifications (Kay et al., 2014; Leydesdorff et al., 2014): the assumption that all categories in the map belong to the same ontology. A way forward is to develop separate mappings for each of the multiple ontologies encountered. One example are the mappings created with Medical Subject Headings (MeSH) where emerging technologies were analysed in various ontologies according to the various branches of the hierarchical classification: Diseases; Chemicals, Techniques and Equipment (Leydesdorff et al., 2012). The attempt to describe these entanglements through heterogenous networks are sometimes useful for academic purposes (Bourret et al., 2006), but they are generally difficult to understand. Instead, we propose that layered structures are easier to interpret. Adopting this perspective does not mean to deny the entanglement between the various layers. It is an attempt to make analysis easier to understand (i.e. to decompose something into its elements in order to study it).

**Fig. 9:**
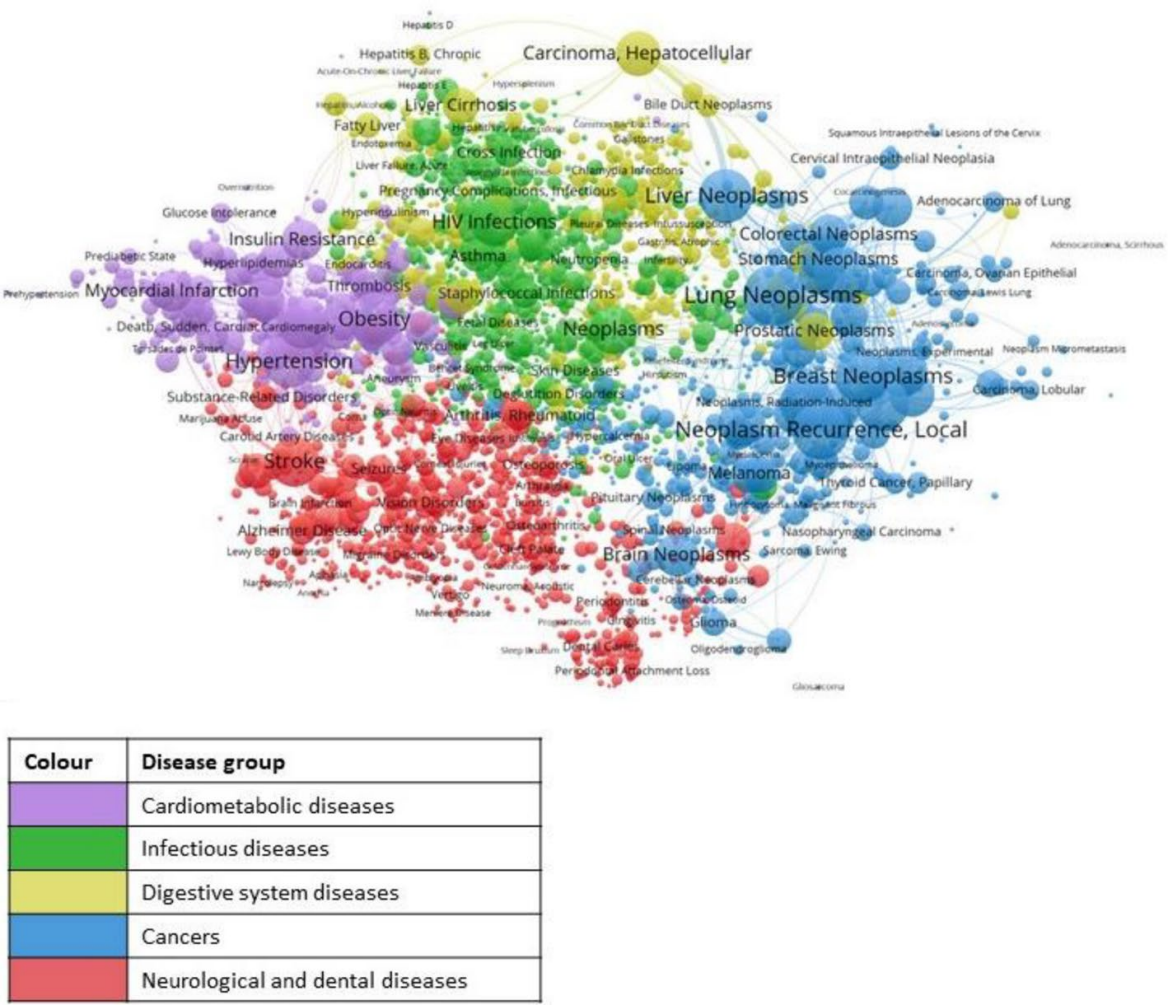
MeSH co-occurrence network. The colours of the node clusters represent different groups of diseases, as specified in the key. Source: Coburn (2024) (see also Coburn et al. (2024))

Such approach has been used for example, to create a map of diseases based on MeSH, which is used to explore to which extent research grants have ‘spillovers’ beyond the diseases targeted by the grant according to the grant application (Coburn, 2024; Coburn et al., 2024), as shown in Fig. 9. Let me observe that while significant changes in classifications (from one disciplinary to another disciplinary classification) may not result in a substantially different map (as shown in (Rafols & Leydesdorff, 2009)), changes in ontology from one based on disciplines to one based on diseases often produces a radically different structure.

Similarly to Fig. 9, maps based on methods and techniques could be developed using the E branch of the MeSH hierarchy, related to methods. The expectation is that a map of methods would have a very different logic. De Solla Price (1984) observed that papers focused on instrumentation and research materials had different patterns in citation maps as they spread over different cognitive spaces. Hladík and Renisio (2025) have indeed shown that a ‘methods and materials’ axis constitute the third principal component of a global classification of science, and that it produces a very different epistemic space in comparison to the combination of first axis (culture vs. nature) and second axis (life vs. non-life).

How could the map approach obtained using the different branches of the medical hierarchy be generalised to areas that don’t have such multi-perspective tagging? The answer lies in the semantic methods that are transforming scientometrics: new classification methods based on machine learning offer the opportunity of tagging and developing classifications based on specific ontologies, tailored to provide the ontological perspectives which seems most relevant to the questioned posed.

Instead of using top-down conventional disciplinary categories, Bovenzi et al. (2022) created a classification model based on the categories of the 25 panels of the European Research Council (ERC), by training the model with the publications produced by the projects in each of the panels. The method succeeds at classifying ERC projects to panels with an accuracy estimated around at 94% to 99%, depending on the panel.

Even more important is the possibility of providing classifications based on ontologies which different from disciplines or conventional scientific classifications. Various indexing methods have been developed for automatically tagging publications with MeSH based on training (You et al., 2021) (some of them have been found to be useful, but not very precise, according to Moore et al. (2024)). These new classification methods may be particularly helpful to classify according to logics derived from specific policy-needs for which classifications are problematic or non-existent. For example, health research can be classified in terms four key categories: basic, translational, clinical research, and public health research, based on training sets for each of these categories produced by experts (Durán-Silva et al., 2024). This effort to classify biomedical research has been tried since the 1970s (Narin et al., 1976), but that has always remained a major challenge (Boyack et al., 2014a, 2014b).

Similarly, publications or grants can be classified into selected research priorities specific to a territory such as the EU-supported Regional Innovation Smart Specialisation Strategies (RIS3) after training a classification model (Fuster et al., 2023). This allows to analyse a whole regional portfolio according to perspective provided by the tailored priorities according to their top-down classification, which can be contrasted to taxonomic structures that appear with bottom-up methods (Fuster et al., 2020). A particularly striking example of machine-learning classification is its use for analysing public values such as “security and safety”, “privacy and data security” or “transparency in decision making” over a set 150,000 set of patents (Pelaez et al., 2024). Given that in our study of mental health the policy question was posed in terms of categories such as prevention, healthcare services, biomedical research, brain research, social determinants, diagnosis and treatment, we could have used a similar machine learning algorithm.

## Conclusions

In this article, I have reflected upon Leydesdorff’s major work on science mapping, highlighting his contributions to journal and global science maps, his use of mappings across multiple analytical dimensions, and his critique to unreflective science mapping.

Leydesdorff’s (broad brushstroke) approach to journal maps and the global map of science have proven surprisingly robust in the sense that the same large scale epistemic structures are obtained with a variety of methodological choices, as claimed by Klavans and Boyack (2009) and as shown by more recent studies such as Hladík and Renisio (2025). However, the expectations that new algorithmic methods would produce a clean hierarchical structure of science have not been accomplished: the evidence suggests that there is not a “natural” structure of science. As shown by the case studies on invasion biology and mental health above, the logics of the epistemic groupings are contingent on methodological choices and they become increasingly more difficult to understand as the size of epistemic categories is reduced.

The good news is that machine learning techniques allow now to produce classifications and mappings of science based on the logics of specific epistemic lenses or units (e.g. on problem orientation, on territorial priorities, on public values Durán-Silva et al., 2024; Fuster et al., 2023; Pelaez et al., 2024)), which hitherto could only be applied with very special and costly ontologies such as PubMed’s MeSH (Coburn et al., 2024). Instead of looking for “accurate” portrayals of science, we should recognise that different perspectives will produce different landscapes. Transparency in the data and the methods is also important, as argued by the *Barcelona Declaration on Open Research Information*,^8^ so that it is possible to scrutinise and understand the perspectives used in a certain analysis.

Now, the choice of ontologies and associated methods should depend on how useful they are to answer the questions asked by the analysts. Besides Foucaul’t laugh on Borges’ classification, insights from science and technology studies on classification and ontologies by Mary Douglas, Geoffrey Bowker and Annemarie Mol point in the same direction. A key issue, then, for science mapping is to develop a methodological approach that can relate specific ontologies to certain sociological questions.

In a historical moment in which the sciences are (even more) strongly intertwined with societal and economic forces, questions on how science evolves in relation to societal demands and needs may be better posed with ontologies expressing these demands and needs, rather than internalist ontologies (such as disciplinary). For example, in health in relation the disease categories that relate to burden (Yegros-Yegros et al., 2020) or in related to public values (Pelaez et al., 2024). These ontologies and associated mappings can visualize the alignments and misalignments between a given social aspect and research agendas (Ciarli & Rafols, 2019).

However, this awareness of the need to develop ontologies that respond to social demands and are ‘the right tools for the job’ is not actually what we are witnessing in a lot of computational social sciences. In her book on digital sociology Nortje Marres (2017, p. 187) warns us that “much computational social science projects simply go along with whatever ontology, epistemology or methodology is wired into the platforms, packages or tools they use to capture, analyse and visualize data, without querying whether and how they are appropriate to the research project at hand”.

Marres’ warning echoes the message that Leydesdorff has been telling us since the 1980s with regards to science mapping:

The emphasis in these policy-oriented studies has been on [questionable] validation of the outcomes of the models. Less attention has been paid to the methodological decisions which precede the model building (…) I will argue that precisely some of these methodological decisions, (…) have been basically wrong, and that therefore the co-citation maps in their current form (…) do not represent or represent only very partially "the structure and the dynamics of science". (Leydesdorff, 1987, p. 296)

Perhaps Leydesdorff was (and perhaps I am) overstating the problems of past and present science maps, though we are not alone (Hicks, 1987, 1988; Rip, 1997)). In any case, it is of justice to recognise that Leydesdorff’s critiques, with their unusual combination of affable laugh and irritation, of generosity and impatience, have been important for scientometrics to keep the methodological and ethical rigour, and the engagement with theory, in the face of policy and market rewards that might have driven others to more mundane profits and honours.

40 years have passed since Leydesdorff questioned the methodology of the Institute of Scientific Information (ISI) which claimed to produce a comprehensive and faithful structure of science. The critique has been confirmed: there are some stable structures in science, but there is not a single natural structure. From this vantage point and given the game-changing opportunities that semantic analysis with language models offer, I have proposed here to espouse a shift from a universal view of science maps (with a unique natural structure) to a multiverse view of knowledge mappings that is rooted on the perspectives of potential beneficiaries of science. Keeping this plurality of options with multiple ontologies is particularly relevant for the radical critique of science proposed by Boltanski – and for those of us that think that we need different types of science (and of knowledge) in our highly troubled and unjust world.

## Footnotes

1 I borrow this adjective from Paul Wouters’ tribute to Leydesdorff in the S&T Indicators Conference in 2023.

2 See the full list of contributors: https://www.leydesdorff.net/qss.2020/index.htm If you forgive me the indiscretion, in this effort at bridge building we still witnessed (still in 2020!) in some of the responses to the reviewers, some sparkles from the old sectarian fights between Mertonians and constructivists!

3 I have argued that understandings of interdisciplinarity differ across disciplines, sectors or policy domains. Therefore, a meaningful scientometric operationalisation needs a high degree of contextualization; in consequence general indicators are usually problematic. See: https://www.leidenmadtrics.nl/articles/on-measuring-interdisciplinarity-from-indicators-to-indicating

4 http://www.leydesdorff.net/overlaytoolkit/

5 See https://www.leidenranking.com/information/fields.

6 See https://www.cwts.nl/projects/past-projects/mapping-health-and-well-being-research

7 Image by J-C. Benoist, Wikimedia Commons. https://commons.wikimedia.org/wiki/File:Dualite.jpg

8 https://barcelona-declaration.org/
